# Standardizing Vegetation Size Measurement in Native Left-Sided Infective Endocarditis Using Artificial Intelligence

**DOI:** 10.3390/jcm15155806

**Published:** 2026-07-24

**Authors:** Daniel Pinilla-García, Gonzalo Cabezón-Villalba, Luis Llamas-Fernández, Carlos González-Juanatey, Juan Carlos López-Azor, Carmen Olmos, Chiara Pidone, Manuel Anguita-Sánchez, Luis Martínez-Dolz, Itziar Gómez-Salvador, Alejandro Manuel López-Pena, Noemí Ramos-López, Daniel Gómez-Ramírez, Victoria Delgado, Juan C. Castillo-Domínguez, Raquel Ladrón, José Francisco Gil, María de Miguel, Teresa Sevilla, Ana Revilla-Orodea, Javier López, J. Alberto San Román, Carlos Baladrón

**Affiliations:** 1 Cardiology, Hospital Clínico Universitario de Valladolid, 47003 Valladolid, Spain; gonzavilla10@hotmail.com (G.C.-V.); lllamas@icicor.es (L.L.-F.); itziargs@gmail.com (I.G.-S.); ralaab90@gmail.com (R.L.); josefgilfer@gmail.com (J.F.G.); mdemiguelalava@gmail.com (M.d.M.); tereseru@gmail.com (T.S.); arevillaorodea@gmail.com (A.R.-O.); javihouston@yahoo.es (J.L.); asanroman@secardiologia.es (J.A.S.R.); cbaladron@icicor.es (C.B.); 2 Instituto de Investigación Sanitaria de Valladolid (IBioVALL), 47010 Valladolid, Spain; 3 Centro de Investigación Biomédica en Red de Enfermedades Cardiovasculares (CIBERCV), 28029 Madrid, Spain; manuelanguita@secardiologia.es (M.A.-S.); luismartinezdolz@gmail.com (L.M.-D.); juanc.castillo.dominguez.sspa@juntadeandalucia.es (J.C.C.-D.); 4 Cardiology, Hospital Universitario Lucus Augusti, 27003 Lugo, Spain; carlos.gonzalez.juanatey@sergas.es (C.G.-J.); alejandro.manuel.lopez.pena@sergas.es (A.M.L.-P.); 5 Cardiology, Hospital Universitario Puerta De Hierro Majadahonda, 28222 Madrid, Spainnoemi.rl92@gmail.com (N.R.-L.); 6 Heart Failure and Inherited Cardiac Diseases, Cardiology, Hospital Universitario Puerta De Hierro Majadahonda, 28222 Madrid, Spain; 7 Cardiology, Hospital Clínico San Carlos, Instituto de Investigación Sanitaria del Hospital Clínico San Carlos (IdISSC), 28040 Madrid, Spaindanielgomezramirezmd@gmail.com (D.G.-R.); 8 Institut de Recerca Germans Trias i Pujol, 08916 Badalona, Spain; chiara.pidone22@gmail.com (C.P.); videlga@gmail.com (V.D.); 9 Department of Clinical and Molecular Medicine, Sapienza University of Rome, 00185 Rome, Italy; 10 Hospital Universitario Reina Sofía Cardiología, 14004 Córdoba, Spain; 11 Department of Medical and Surgical Sciences, Universidad de Córdoba, 14014 Córdoba, Spain; 12 UGC Cardiología, Inst. Maimonides de Investigación Biomédica de Córdoba, 14004 Córdoba, Spain; 13 Cardiology, Hospital Universitario y Politecnico La Fe, 46026 Valencia, Spain; 14 Center of Comparative Medicine and Bioimaging, Institut de Recerca Germans Trias i Pujol, 08916 Badalona, Spain

**Keywords:** endocarditis, artificial intelligence, echocardiography, vegetation, measurement standardization

## Abstract

**Background/Objectives**: Vegetation length is a guideline-endorsed criterion for surgery in left-sided infective endocarditis (LSIE). However, its measurement is highly variable with crucial implications for decision making. A standardized measurement system would not only facilitate decision making in these patients, but also cutoff-point optimization for improving clinical guidelines. For this purpose, this work introduces an Artificial Intelligence (AI)-based system capable of extracting vegetation length from standard transesophageal echocardiography (TEE). **Methods:** Five echocardiographers independently measured the vegetation length of 76 vegetations from 67 consecutive patients with LSIE using offline TEE images. The AI-based system was trained on a multicenter registry with 353 patients with LSIE, comprising 282,096 echocardiographic frames annotated by an independent expert. This system was applied to measure vegetation length as an independent observer. The variability and correlation between operators and the system were assessed. **Results**: Lin’s concordance correlation coefficient between the AI-based system and the mean of the measurements obtained by the echocardiographers was 0.74, comparable to the coefficient obtained between the echocardiographers themselves. Bland–Altman analysis showed a mean difference of −1.0 mm between the AI-based system and the mean of the measurements obtained by the echocardiographers. **Conclusions**: The AI-based system for vegetation measurement demonstrates a high level of correlation and agreement with experts, similar to the concordance between human operators themselves. This level of agreement suggests that the method has the potential to reduce inter-operator variability in the measurement process. Whether AI-based vegetation size improves embolism prediction has to be further investigated.

## 1. Introduction

Accurate quantification of vegetation size in left-sided infective endocarditis (LSIE) is critical for risk stratification and surgical decision-making. Current clinical guidelines of both the American Heart Association and the European Society of Cardiology emphasize maximal vegetation length as a key parameter in determining the need for early surgery, particularly for embolism prevention [1,2]. The 2020 AHA/ACC guidelines recommend consideration of early surgical intervention for mobile left-sided vegetations exceeding 10 mm in length, irrespective of lesion severity or operative risk profile (Class IIb recommendation) [3]. The 2023 ESC guidelines further recommend urgent surgery for aortic or mitral valve vegetations greater than 10 mm in the presence of severe valvular stenosis or regurgitation and low operative risk (Class IIa recommendation), or for any vegetation exceeding 15 mm [2]. However, the methodological foundation for this recommendation remains weak [4,5,6,7]. Vegetation size is subject to substantial measurement uncertainty. Echocardiographic evaluation of vegetation size has intrinsic limitations: vegetations are often highly mobile, irregular in shape, of diverse echogenicity, and sometimes multiple, making precise and consistent measurement inherently difficult. Operator-dependent factors, such as image interpretation and frame selection, further increase variability. This interobserver inconsistency not only limits clinical reliability but also introduces statistical error into analyses.

Importantly, variability in the measurement of vegetation size may have a critical impact on disease management in LSIE [8]. In this setting, a size-based threshold determines whether to proceed with urgent surgery or conservative management, so that even small measurement differences may lead to different therapeutic decisions [8]. This fact challenges the validity of using vegetation size as an independent and decisive parameter for surgery, and explains why embolism prevention is a controversial indication not always implemented in clinical practice. 

In response to this unmet clinical need, we developed an AI-based system capable of automatically detecting and measuring vegetations in transesophageal echocardiography (TEE). By reducing interobserver variability in the measurement process, this tool may enhance the reliability of vegetation size as a prognostic and therapeutic criterion. This work aims to evaluate the performance of this algorithm in comparison with conventional measurements performed by expert echocardiographers.

## 2. Materials and Methods

In this study, the measurements of the maximum length of vegetations obtained by five echocardiographers were compared with those obtained by an AI-based system developed by our group. To perform this analysis, TEE data from 76 vegetations in 67 consecutive patients diagnosed with definite LSIE according to the modified Duke criteria [9] at a single tertiary care center were analyzed. The AI-based system was trained using annotated data from the EMBOENDO dataset, a multicenter echocardiographic registry comprising 353 patients with confirmed diagnoses of left-sided IE, retrospectively collected between 2014 and 2025. The dataset, consisting of approximately 282,096 images, underwent manual annotation by a sixth operator, distinct from the personnel responsible for the vegetation assessments, over a two-year period. Vegetations were defined as abnormal intracardiac masses, oscillating or not, adherent to a valve or other endocardial surface, with an independent and erratic movement. Patients with mechanical or bioprosthetic valves were excluded to avoid confounding due to prosthesis-related imaging artifacts and measurement difficulties. Cases involving right-sided endocarditis or device-related infection were not considered given that vegetation size is not considered a criterion for surgery. Demographic and baseline clinical data for the study population are presented in Table 1.

**Figure 1:**
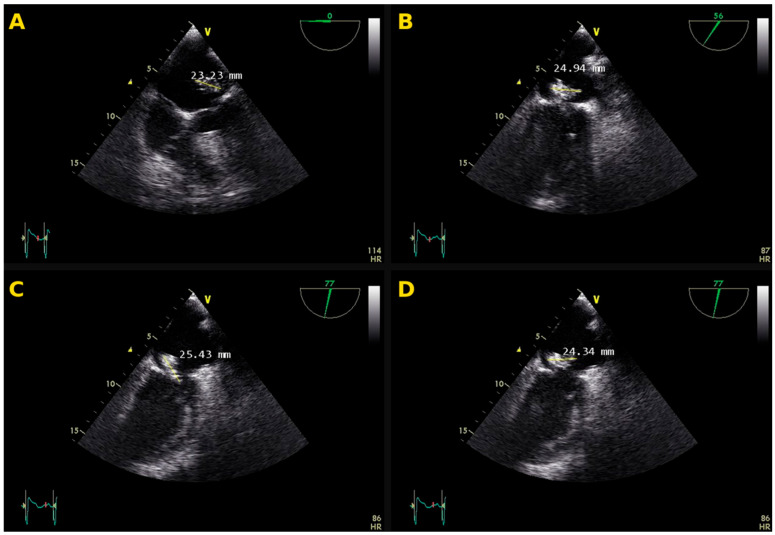
Manual diameter measurement (yellow line) performed by an imaging expert in several representative frames from a study. The expert performs the measurement in different frames of each cine loop in which the vegetation is considered to be largest. In this case, the expert performed measurements in three different views (**A**–**C**) at imaging plane angles of 0°, 56°, and 77°, respectively, and in two different frames of view (panels (**C**,**D**)), obtaining a final vegetation measurement of 25.43 mm in panel (**C**), as it corresponded to the largest of all measurements.

All included patients underwent standardized 2D-TEE echocardiographic longitudinal or three-chamber views (excluding x-plane and color Doppler) captured in standard-of-care exams. Five echocardiographers, all affiliated with a high-volume imaging unit specializing in endocarditis, independently measured the vegetation length of 76 vegetations as typically performed in routine clinical practice. Vegetation length was measured using its maximal longitudinal diameter, as recommended by clinical practice guidelines. To assess the real variability of clinical practice, each echocardiographer independently selected the cine loops and frames for measurement from all available acquisitions for each patient and was blinded to the measurements and loop/frame selections of the other echocardiographers. For each cine-loop, frames were selected at points in the cardiac cycle where the vegetations appeared most well-defined. Measurements were taken in at least two orthogonal planes, and the largest value observed was recorded as the maximal longitudinal diameter. The entire process requires approximately 10 min, depending on the vegetation type, the complexity of its measurement, the number of vegetation instances, and the number of loops available for the patient. Figure 1 illustrates a manual diameter measurement in several representative frames from a study.

The high quality of the TEE images minimizes the impact of image noise, which therefore does not constrain the system’s measurement performance. The images were acquired using Vivid E95 General Electric ultrasound systems with 6VT probes (GE Vingmed Ultrasound AS, Horten, Norway) and a transducer frequency of 5 MHz. The measurements were performed offline using EchoPAC software, version 206 (GE Vingmed Ultrasound AS, Horten, Norway).

In parallel, each patient’s TEE imaging set was processed using an AI-based system developed for the automatic detection and measurement of vegetations. The system processes all available loops for a given patient, yielding a measurement of the vegetation length of the detected vegetations within each loop, and subsequently selecting the largest value across all loops as the final output. Importantly, whenever the same set of videos is processed, the system consistently produces identical results. The algorithm identifies the valve on which each vegetation is located and, when vegetations are detected on both valves, provides individual measurements for each, specifying the corresponding valve. The complete measurement process for a single cineloop typically requires between 3 and 15 s, with duration primarily contingent upon the number of frames comprising the cineloop. In cases where a patient presents with a substantial number of cineloops or particularly extended sequences (120–200 frames per loop), the total processing time may range from one to two minutes, proportionally dependent on both the frame count per cineloop and the total number of cineloops acquired per patient.

### 2.1. Algorithm

The algorithm comprises three deep-learning modules: a frame-level vegetation detection model, a segmentation model that delineates vegetation contours, and a tracking algorithm to integrate information of separated frames across a cine loop. 

The detection model is a YOLOv12 network that takes a single TEE frame as input and outputs the bounding-box coordinates of the detected vegetations [10]. Relative to our previous model, its performance was improved by expanding the training dataset to a larger patient cohort.

**Figure 2:**
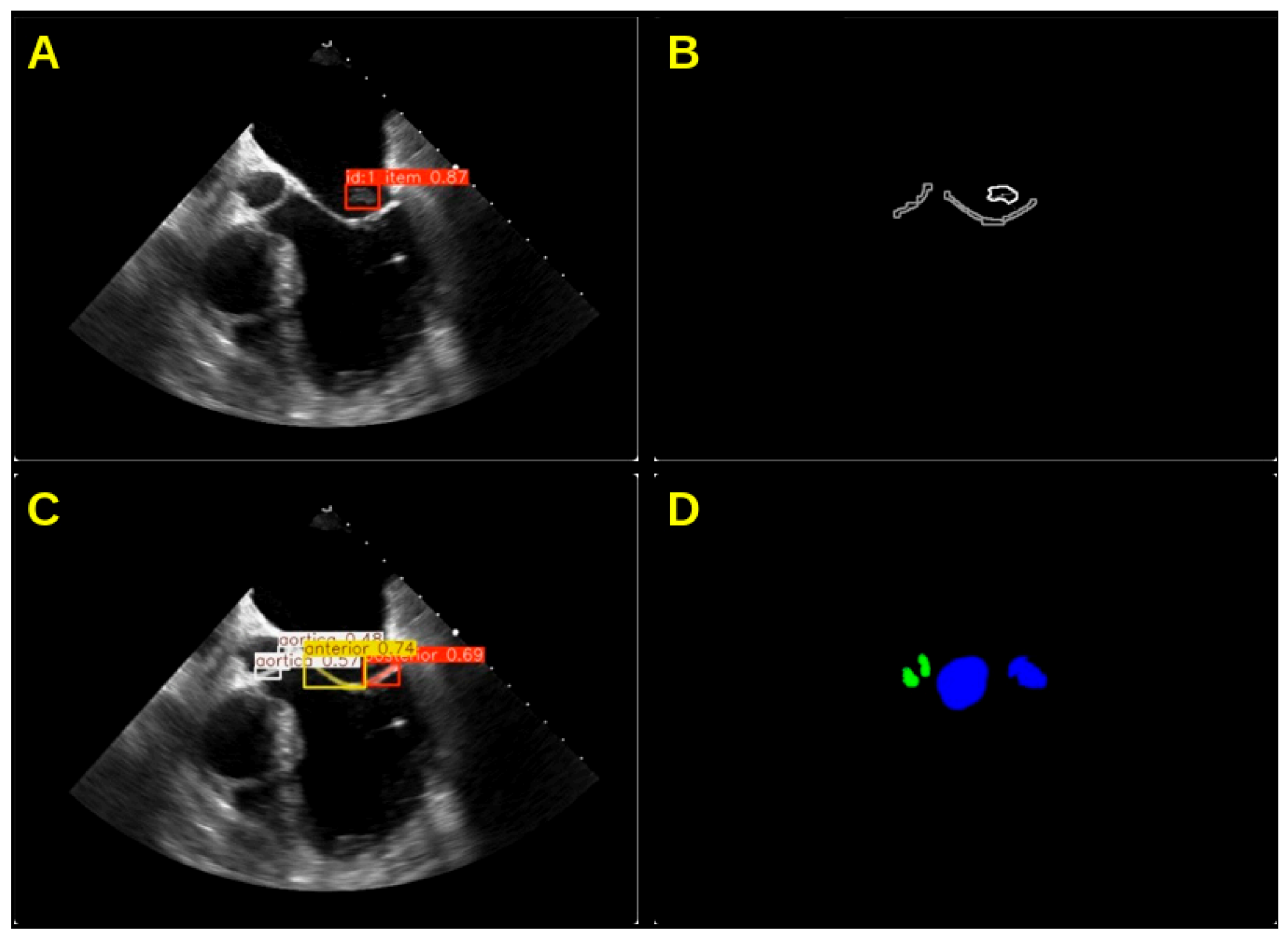
Example output of the vegetation detection and segmentation algorithm. (**A**): Bounding box generated by the detection model. (**B**): Final vegetation and valves contours showing the maximum vegetation diameter in the corresponding frame. (**C**): Valve masks generated by the valve segmentation model. (**D**): Heatmaps generated during the intermediate stages of the algorithm.

The segmentation model delineates the contour of each detected vegetation and estimates its length, defined as the distance between the two farthest points on the contour. The tracking algorithm includes a valve segmentation model that places detected vegetations in their anatomical context and enables distinct identifiers to be assigned to each vegetation when more than one is present within a cine loop. This step is critical because multiple vegetations may coexist in a single TEE sequence, and the algorithm must reliably track each vegetation despite intermittent visibility across frames. The confidence thresholds were set at 0.50 for vegetation detection, 0.15 for vegetation segmentation, and 0.31 for valve segmentation. Figure 2 shows representative images of the algorithm’s operation throughout a cine loop: the top-left panel (Figure 2A) shows the bounding boxes produced by the vegetation detection model; the top-right panel (Figure 2B), the contours generated by both the valve and vegetation segmentation models; the bottom-left panel (Figure 2C), the masks from the vegetation segmentation model; and the bottom-right panel (Figure 2D), the movement “heatmaps” of the two valves. A full description of this algorithm is provided in Appendix C.

**Figure 3:**
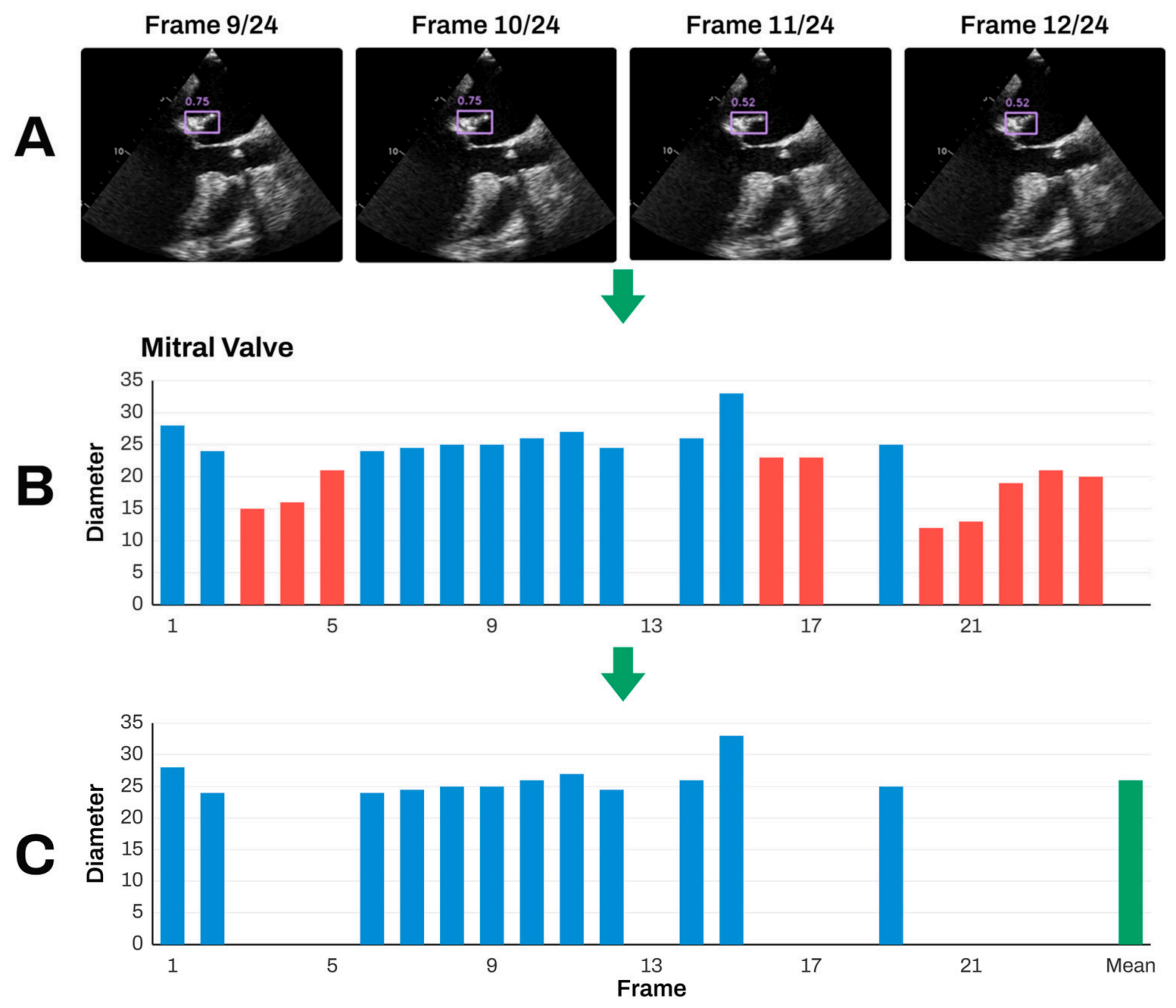
Stepwise process for automated vegetation measurement. (**A**) Frame-by-frame detection of the mitral valve vegetation across consecutive frames (Frames 9/24–12/24), with bounding boxes and confidence scores displayed for each detection. (**B**) Diameter measurements obtained for each detected frame. Lower diameter measurements (red bars), typically arising from submaximal visualization of the vegetation profile, are excluded from the final aggregation, whereas higher measurements (blue bars) are retained to estimate maximal vegetation size. (**C**) Final vegetation diameter estimation, calculated as the mean of the upper half of the retained diameter values (green bar), yielding a robust final measurement.

For each patient, the algorithm processes all available cine loops and selects the one yielding the maximal measurement. Vegetations, initially detected on a frame-by-frame basis (Figure 3A), are then integrated throughout the cine loop by tracking anatomical structures over time and associating each vegetation with the valve to which it is attached. Detections that cannot be linked to a consistent anatomical structure across consecutive frames are considered spurious and discarded. For tracked vegetations, the algorithm identifies the two most distant points on each contour, yielding the maximum vegetation diameter for each vegetation in every frame (Figure 3B). Finally, to obtain a robust estimate of maximal vegetation size and reduce noise, frame-wise diameter measurements were ranked, and the final diameter was computed as the mean of the upper 50% of observations. This approach reduces the impact of transient underestimations while preserving information from frames depicting the vegetation near its maximal extent (Figure 3C).

When two vegetations are detected on the same valve in the same frame, the one with the larger diameter is selected, on the premise that, in the absence of other clinical indicators, the largest vegetation is the most relevant parameter for surgical decision-making.

### 2.2. Training Dataset

The vegetation detection and segmentation models were trained using annotated data from the EMBOENDO dataset, a multicenter echocardiographic registry comprising 353 patients with confirmed diagnoses of left-sided IE. The valve segmentation model was developed using a dataset consisting of TEE acquisitions from 77 patients without LSIE. These individuals were randomly selected from patients who underwent TEE within the past five years at the coordinating institution, none of whom had clinical or echocardiographic evidence of LSIE. This approach ensured that the model learned valve morphology independently of pathological features. 

These 353 patients were divided into two subsets: 313 patients were used to fit the model weights and 40 to optimize the training process (to select the epoch at which to stop the training). The same partition was applied to all models. The valve segmentation model was developed using a separate cohort of 77 patients without IE divided into two subsets: 62 patients to fit the model weights and 15 patients to optimize the training process.

The 67 patients in whom vegetation length was measured by the five echocardiographers and by the AI-based system constitute an entirely independent cohort: none of their images were used at any stage of model development, including weight fitting, epoch selection, or hyperparameter tuning. We note that this independence is at the patient level rather than at the center level, as the tertiary center from which this cohort was drawn also contributed patients to the multicenter EMBOENDO registry; no individual patient, however, appears in both datasets.

All subsets were partitioned on a per-patient basis, ensuring that all images from a given patient were confined to a single subset. This approach was adopted instead of splitting by loop or frame in order to prevent data leakage. Including images from the same patient in both sets could allow the model to memorize patient-specific features, thereby compromising its ability to generalize. Such leakage would likely lead to a significant overestimation of model performance during validation.

Annotations were performed with the Visual Object Tagging Tool (VoTT) by one operator distinct from the five echocardiographers who participated in the measurement study and were blinded to their assessments, over a two-year period. Vegetations were annotated according to the definition above, and their presence and location had been established independently of the annotation process: each vegetation was documented in the clinical report of the corresponding echocardiographic study and recorded in the EMBOENDO database by a physician at the contributing center. 

Given the size of the dataset (282,096 images), an iterative semi-automatic procedure was adopted: a model trained on the first 149 patients generated candidate bounding boxes for successive batches of 15 patients, which were reviewed and corrected image by image before being used to retrain the model, until all 353 patients had been annotated. Every candidate label was inspected; none was accepted without review. The reduction in annotation time across iterations reflected a decreasing number of required corrections rather than a less exhaustive review. The same preprocessing used during training was applied during annotation, so that annotator and model operated on identical inputs. 

As explained, the proposed framework operates at the patient level by integrating the outputs of the detection and segmentation models. However, since each model was developed using individual echocardiographic images as training samples, its performance was evaluated at the image level using the optimization dataset. Accordingly, mAP50, mAP50–95, and the F1 score are reported for each model in Table 2. These metrics were obtained on the optimization set, which was used to select the stopping epoch; they should therefore be interpreted as an upper bound rather than as held-out performance.

Although the valve segmentation metrics were comparatively low, the corresponding detection performance was satisfactory (F1 = 0.75, mAP50 = 0.70, and mAP50–95 = 0.24). As the valve segmentation model is intended solely to identify the anatomical location of the valves, without requiring precise delineation of the valve leaflets, these performance levels are adequate for its role within the overall framework. Furthermore, this model received the least development effort among the three components of the pipeline, and its performance is expected to improve substantially with the availability of a larger set of manually annotated images.

Nevertheless, these metrics alone do not fully reflect the overall clinical performance of the proposed framework, which depends on the combined contribution of all three models. Consequently, a relatively modest performance for one of these metrics may still be sufficient if the corresponding model fulfills its intended role within the complete algorithm and does not compromise the final patient-level decision.

### 2.3. Statistical Analysis

Categorical variables are reported as frequencies (*n*) and percentages, while continuous variables are expressed as mean values with standard deviations. To evaluate variability between measurements obtained by each human operator and the AI-based system, both absolute and relative differences from the mean of the measurements were calculated. Several complementary statistical approaches were applied to assess agreement and reliability. Overall reliability was quantified using the intraclass correlation coefficient (ICC) [11], which estimates the proportion of total variability attributable to true differences between subjects rather than to measurement error. Absolute agreement between pairs of methods was evaluated using Lin’s concordance correlation coefficient (CCC) [12], which combines measures of precision and accuracy and penalizes systematic bias. Bland–Altman plots were generated for each pair of methods to explore agreement in terms of mean bias and limits of agreement. To assess the level of diagnostic concordance in clinical decision-making, maximum vegetation diameters were stratified into three clinically relevant risk categories (<10 mm, 10–14.9 mm, and ≥15 mm), corresponding to established surgical decision thresholds. Inter-observer agreement for all pairwise combinations was evaluated using Cohen’s kappa coefficient (Appendix D). To account for the ordinal nature of the classifications and to penalize larger disagreements more heavily, the quadratic-weighted kappa coefficient was also calculated. Additionally, because Cohen’s kappa is highly sensitive to category prevalence, the positive agreement (specific agreement) was calculated separately for each of the three ordinal categories. Kappa values were interpreted according to the Landis and Koch criteria.

To evaluate differences in continuous measurements of vegetation size (in millimeters) among the five human observers and the artificial intelligence (AI) system, a pairwise leave-one-out approach was employed. Because vegetation size measurements did not follow a normal distribution, as confirmed by the Shapiro–Wilk test, non-parametric methods were used for continuous data analysis. To assess whether systematic overestimation or underestimation existed between each observer and every other observer, as well as relative to the dynamic consensus metrics (the mean and median values of the remaining evaluators), the Wilcoxon signed-rank test for paired samples was performed across all pairwise comparisons. A two-sided *p*-value < 0.05 was considered statistically significant, leading to rejection of the null hypothesis of marginal homogeneity.

All analyses were conducted using R software, version 4.4.2 (R Project for Statistical Computing).

## 3. Results

**Figure 4:**
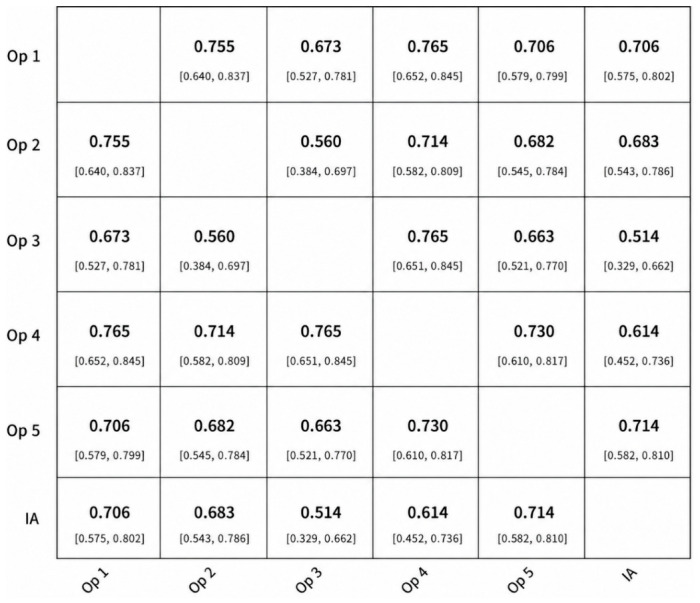
Correlation coefficients among the operators, as well as between the operators and the AI-based system. Similar values can be observed both between the operators and the AI-based system, as well as among the operators themselves.

Table A1, in Appendix A, shows the measurements of the 76 vegetations, taken by both the operators and the system, in 67 patients with LSIE included in the study. Concordance in the assessment of the maximal vegetation length among the operators, as well as between the operators and the AI-based system, was quantified using the CCC, the results of which are presented in Figure 4. 

Compared with the average measurements of the operators, the AI system exhibited an absolute difference of 3.56 ± 4.33 mm and a relative difference of 22.97 ± 17%. Agreement between the two approaches was moderate to good, yielding an ICC of 0.736 (95% CI: 0.612–0.825) and a CCC of 0.736 (95% CI: 0.615–0.828). When the median of the operator measurements was used as the reference instead of the mean, agreement decreased slightly but remained moderate to good (ICC 0.710, 95% CI: 0.573–0.808). Restricting the comparison to the mean of the two most experienced operators yielded a slightly higher agreement, with an ICC of 0.749 (95% CI: 0.629–0.835) and an absolute difference of 3.09 ± 2.50 mm.

**Figure 5:**
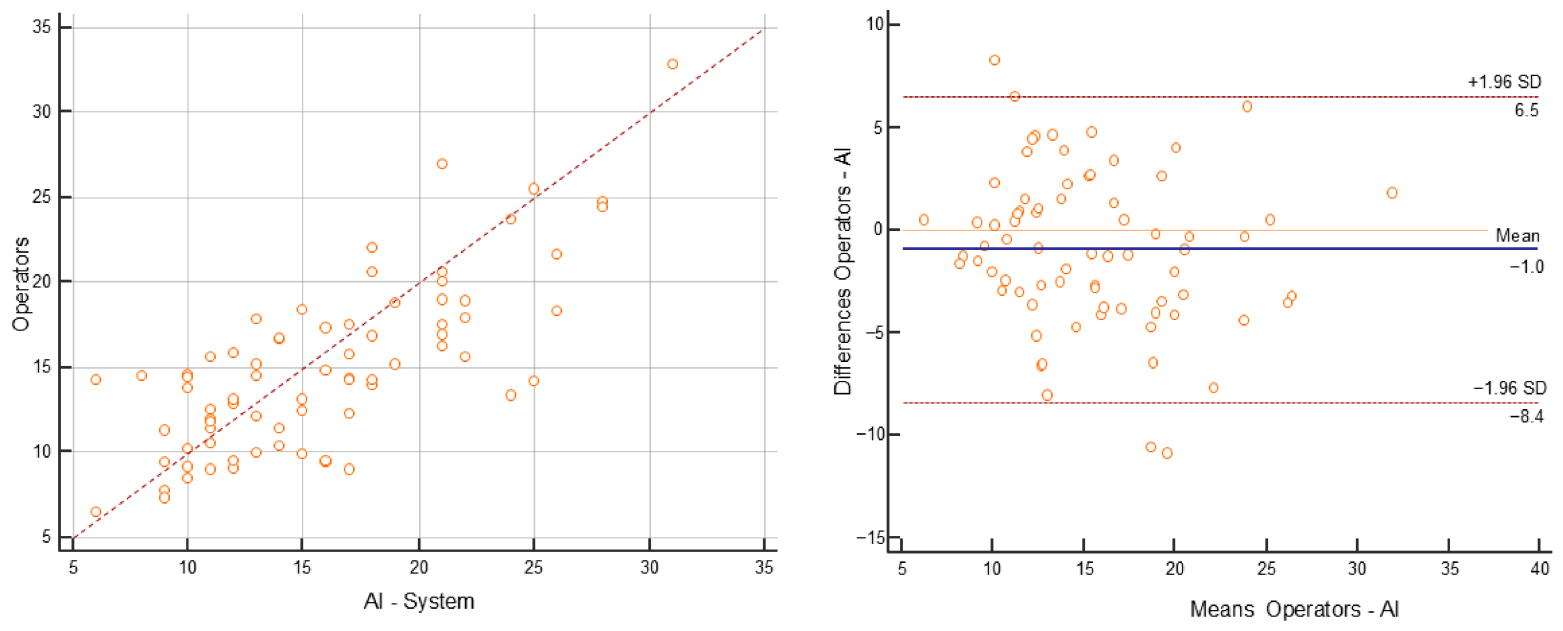
Left to right: scatter plots illustrating the correlations between measurements obtained by the means of the Operators and the AI system. The right image shows Bland–Altman plots evaluating the agreement between measurements obtained by Operators and the AI system. In both panels, each orange circle represents one paired measurement. In the scatter plot, the diagonal line is the line of identity, corresponding to perfect agreement. In the Bland–Altman plot, the solid blue line indicates the mean difference between mean operator measurements and the AI system, and the red dashed lines indicate the upper and lower 95% limits of agreement.

**Figure A1:**
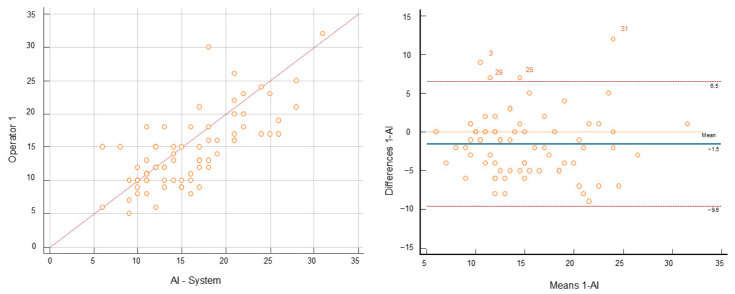
From left to right: scatter plot showing the correlation between measurements obtained by the AI system and Operator 1, and Bland–Altman plot assessing the agreement between measurements obtained by both methods. In both panels, each orange circle represents one paired measurement. In the scatter plot, the diagonal line is the line of identity, corresponding to perfect agreement. In the Bland–Altman plot, the solid blue line indicates the mean difference between Operator and the AI system, and the red dashed lines indicate the upper and lower 95% limits of agreement.

**Figure A2:**
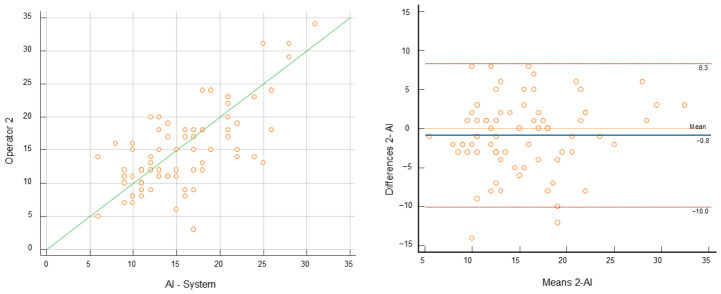
From left to right: scatter plot showing the correlation between measurements obtained by the AI system and Operator 2, and Bland–Altman plot assessing the agreement between measurements obtained by both methods. In both panels, each orange circle represents one paired measurement. In the scatter plot, the diagonal line is the line of identity, corresponding to perfect agreement. In the Bland–Altman plot, the solid blue line indicates the mean difference between Operator and the AI system, and the red dashed lines indicate the upper and lower 95% limits of agreement.

**Figure A3:**
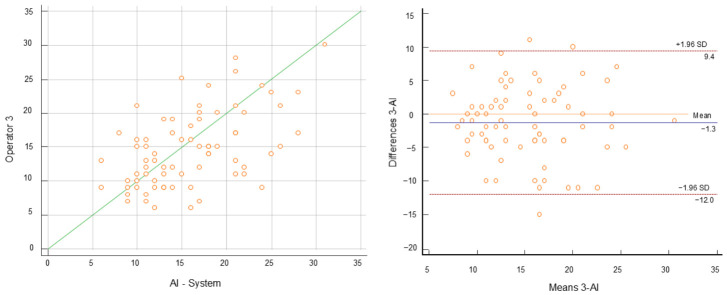
From left to right: scatter plot showing the correlation between measurements obtained by the AI system and Operator 3, and Bland–Altman plot assessing the agreement between measurements obtained by both methods. In both panels, each orange circle represents one paired measurement. In the scatter plot, the diagonal line is the line of identity, corresponding to perfect agreement. In the Bland–Altman plot, the solid blue line indicates the mean difference between Operator and the AI system, and the red dashed lines indicate the upper and lower 95% limits of agreement.

**Figure A4:**
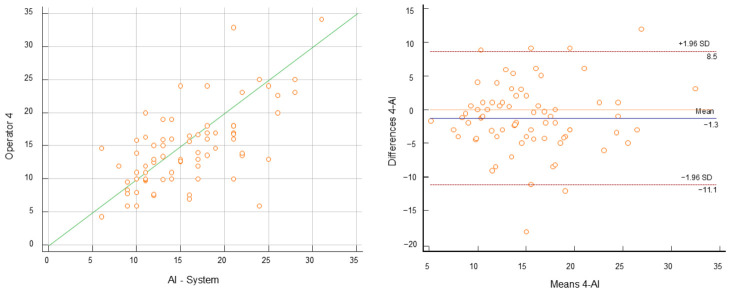
From left to right: scatter plot showing the correlation between measurements obtained by the AI system and Operator 4, and Bland–Altman plot assessing the agreement between measurements obtained by both methods. In both panels, each orange circle represents one paired measurement. In the scatter plot, the diagonal line is the line of identity, corresponding to perfect agreement. In the Bland–Altman plot, the solid blue line indicates the mean difference between Operator and the AI system, and the red dashed lines indicate the upper and lower 95% limits of agreement.

**Figure A5:**
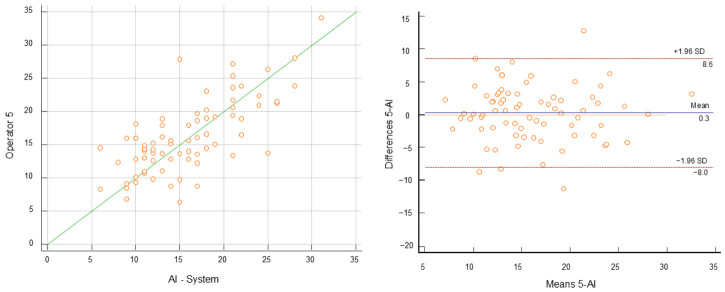
From left to right: scatter plot showing the correlation between measurements obtained by the AI system and Operator 5, and Bland–Altman plot assessing the agreement between measurements obtained by both methods. In both panels, each orange circle represents one paired measurement. In the scatter plot, the diagonal line is the line of identity, corresponding to perfect agreement. In the Bland–Altman plot, the solid blue line indicates the mean difference between Operator and the AI system, and the red dashed lines indicate the upper and lower 95% limits of agreement.

**Figure A6:**
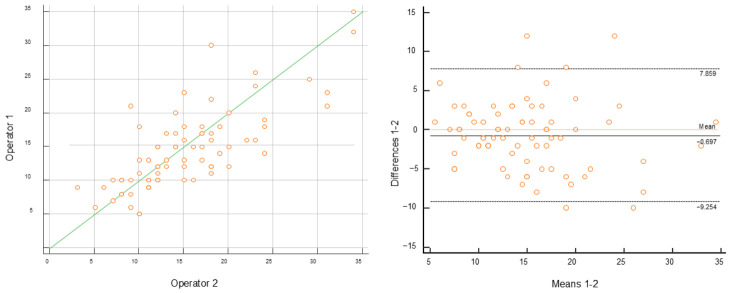
From left to right: scatter plot showing the correlation between measurements obtained by Operators 1 and 2, and Bland–Altman plot assessing the agreement between the measurements obtained by these operators. In both panels, each orange circle represents one paired measurement. In the scatter plot, the diagonal line is the line of identity, corresponding to perfect agreement. In the Bland–Altman plot, the solid blue line indicates the mean difference between Operators.

**Figure A7:**
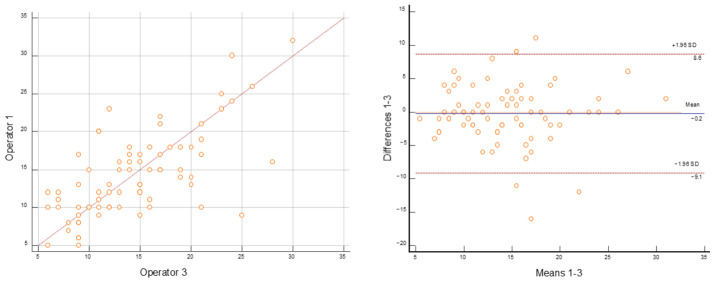
From left to right: scatter plot showing the correlation between measurements obtained by Operators 1 and 3, and Bland–Altman plot assessing the agreement between the measurements obtained by these operators. In both panels, each orange circle represents one paired measurement. In the scatter plot, the diagonal line is the line of identity, corresponding to perfect agreement. In the Bland–Altman plot, the solid blue line indicates the mean difference between Operators.

**Figure A8:**
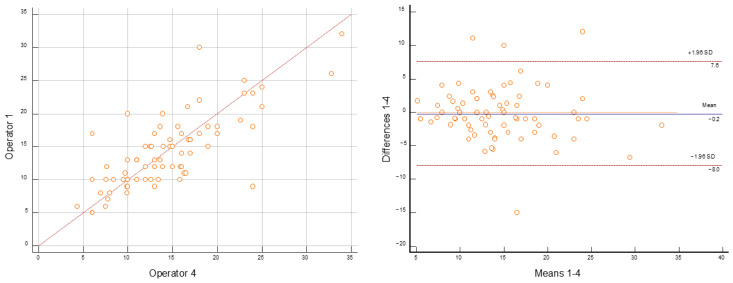
From left to right: scatter plot showing the correlation between measurements obtained by Operators 1 and 4, and Bland–Altman plot assessing the agreement between the measurements obtained by these operators. In both panels, each orange circle represents one paired measurement. In the scatter plot, the diagonal line is the line of identity, corresponding to perfect agreement. In the Bland–Altman plot, the solid blue line indicates the mean difference between Operators.

**Figure A9:**
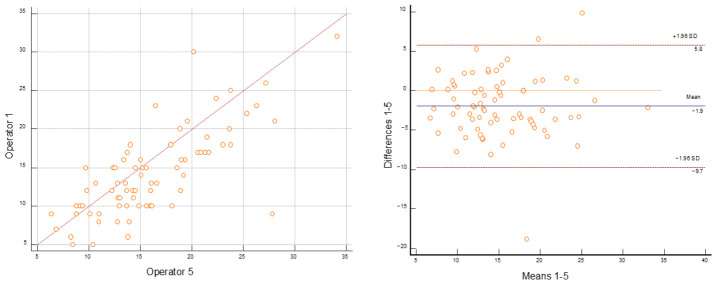
From left to right: scatter plot showing the correlation between measurements obtained by Operators 1 and 5, and Bland–Altman plot assessing the agreement between the measurements obtained by these operators. In both panels, each orange circle represents one paired measurement. In the scatter plot, the diagonal line is the line of identity, corresponding to perfect agreement. In the Bland–Altman plot, the solid blue line indicates the mean difference between Operators.

**Figure A10:**
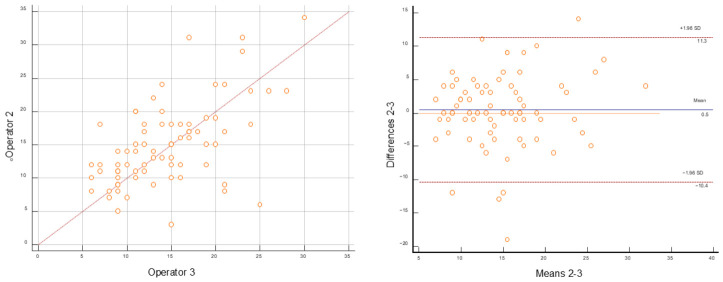
From left to right: scatter plot showing the correlation between measurements obtained by Operators 2 and 3, and Bland–Altman plot assessing the agreement between the measurements obtained by these operators. In both panels, each orange circle represents one paired measurement. In the scatter plot, the diagonal line is the line of identity, corresponding to perfect agreement. In the Bland–Altman plot, the solid blue line indicates the mean difference between Operators.

**Figure A11:**
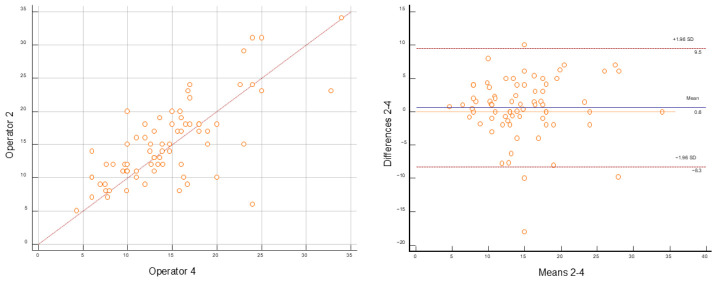
From left to right: scatter plot showing the correlation between measurements obtained by Operators 2 and 4, and Bland–Altman plot assessing the agreement between the measurements obtained by these operators. In both panels, each orange circle represents one paired measurement. In the scatter plot, the diagonal line is the line of identity, corresponding to perfect agreement. In the Bland–Altman plot, the solid blue line indicates the mean difference between Operators.

**Figure A12:**
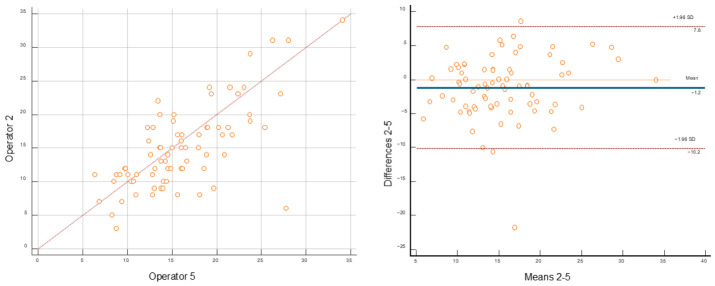
From left to right: scatter plot showing the correlation between measurements obtained by Operators 2 and 5, and Bland–Altman plot assessing the agreement between the measurements obtained by these operators. In both panels, each orange circle represents one paired measurement. In the scatter plot, the diagonal line is the line of identity, corresponding to perfect agreement. In the Bland–Altman plot, the solid blue line indicates the mean difference between Operators.

**Figure A13:**
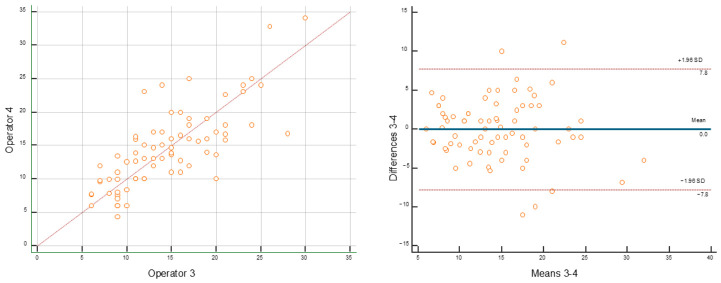
From left to right: scatter plot showing the correlation between measurements obtained by Operators 3 and 4, and Bland–Altman plot assessing the agreement between the measurements obtained by these operators. In both panels, each orange circle represents one paired measurement. In the scatter plot, the diagonal line is the line of identity, corresponding to perfect agreement. In the Bland–Altman plot, the solid blue line indicates the mean difference between Operators.

**Figure A14:**
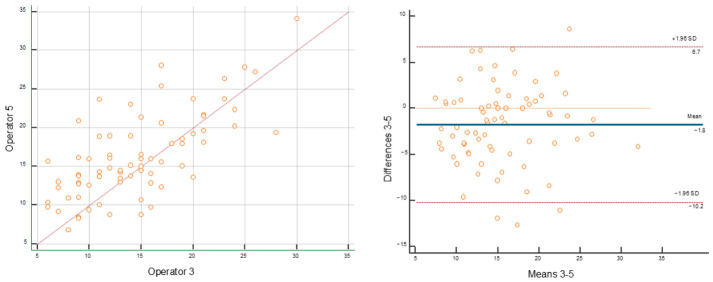
From left to right: scatter plot showing the correlation between measurements obtained by Operators 3 and 5, and Bland–Altman plot assessing the agreement between the measurements obtained by these operators. In both panels, each orange circle represents one paired measurement. In the scatter plot, the diagonal line is the line of identity, corresponding to perfect agreement. In the Bland–Altman plot, the solid blue line indicates the mean difference between Operators.

**Figure A15:**
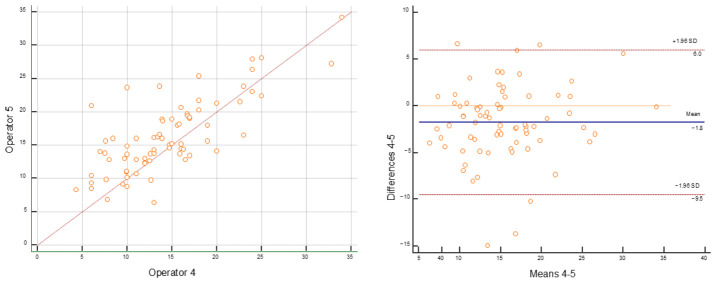
From left to right: scatter plot showing the correlation between measurements obtained by Operators 4 and 5, and Bland–Altman plot assessing the agreement between the measurements obtained by these operators. In both panels, each orange circle represents one paired measurement. In the scatter plot, the diagonal line is the line of identity, corresponding to perfect agreement. In the Bland–Altman plot, the solid blue line indicates the mean difference between Operators.

Figure 5 summarizes the relationship between the AI-based system and operator measurements. The left panel presents a scatter plot illustrating the correlation between mean operator measurements and those obtained by the AI-based system, comparable to the correlation plots in Figure A1, Figure A2, Figure A3, Figure A4, Figure A5, Figure A6, Figure A7, Figure A8, Figure A9, Figure A10, Figure A11, Figure A12, Figure A13, Figure A14 and Figure A15 (left panels) of Appendix B, which depict relationships among operators and between each individual operator and the AI-based system. The right panel presents a Bland–Altman plot evaluating agreement between mean operator measurements and those obtained by the AI-based system, consistent with the agreement patterns shown in Figure A1, Figure A2, Figure A3, Figure A4, Figure A5, Figure A6, Figure A7, Figure A8, Figure A9, Figure A10, Figure A11, Figure A12, Figure A13, Figure A14 and Figure A15 (right panels) of Appendix B, which assess agreement both among operators and between the AI-based system and each individual operator.

In the clinical context, the concordance between the AI-based system and the operators regarding surgical decision-making based on vegetation size was evaluated. Table 3 and Table 4 summarize the agreement between the AI system and the operators in determining the indication for surgery according to the 10 mm and 15 mm vegetation size thresholds, respectively. Taking the operator mean as reference, overall agreement at the 10 mm threshold was 84.7% (61/72), with a sensitivity of 0.950 (57/60) and a specificity of 0.333 (4/12) (Table 3). Regarding the 15 mm threshold, overall agreement was 75.0% (54/72), with a sensitivity of 0.813 (26/32) and a specificity of 0.700 (28/40) (Table 4).

When applying the surgical decision threshold of ≥10 mm, as recommended by the European guidelines, and considering only the two most experienced echocardiographers (T.S. and A.R.), the system’s indication for surgery concurred with at least one of them in 86% of cases and with both in 74%, whereas the inter-expert agreement reached 88%.

Notably, the algorithm successfully detected vegetations in all cases where a suitable view was available; only in two of the 76 cases (entries 2 and 41) was the measurement not performed, because there were no adequate views (only dual-mode displays with a superimposed color Doppler acquisition). The failure rate is therefore 0.026.

Table A2, in Appendix D, reports the agreement between every pair of observers, including the AI-based system, in the classification of vegetations into the three surgical decision categories.

**Figure 6:**
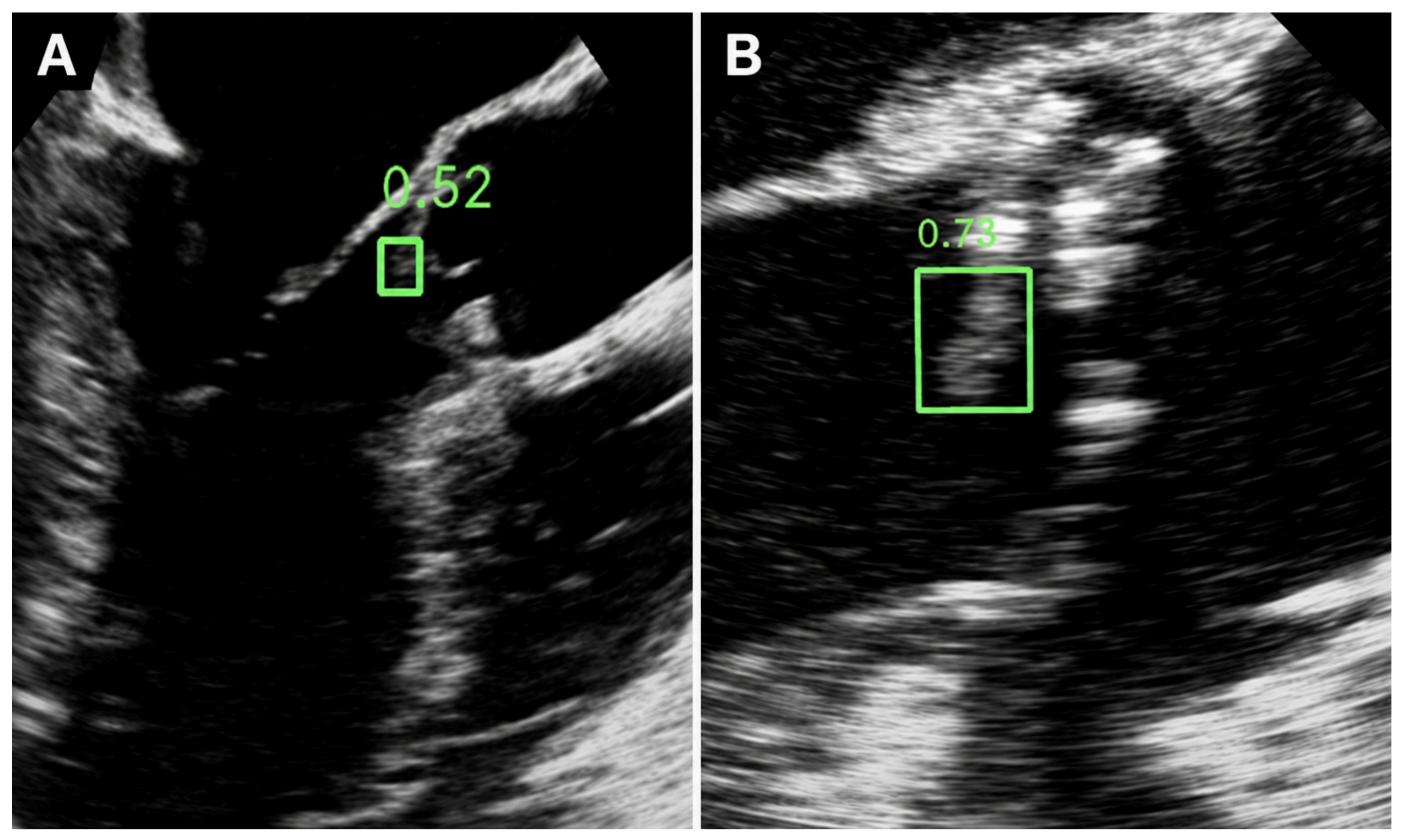
Cases showing the greatest discrepancy between the operators and the AI-based system. Bounding boxes indicate the vegetations identified by the detection model, together with the corresponding confidence score for the displayed frame. (**A**) Patient 3: a homogeneous vegetation whose echogenicity is similar to that of the valve leaflets to which it is attached, making its contour difficult to delineate. (**B**) Patient 29: a more diffuse valvular infection, in which the vegetation comprises regions of differing echogenicity.

The greatest measurement discrepancies between the operators and the system were observed in patients 3 and 29 (Table A1), in whom the boundary between the vegetation and the valve was particularly difficult to define. In both, the algorithm markedly underestimated the vegetation length, placing it below the 10 mm threshold while all five operators measured above it. In Figure 6A, corresponding to patient 3, the vegetation is homogeneous and exhibits echogenicity similar to that of the valve leaflets to which it is attached, making its contour difficult to define. Although the system correctly identifies the location of the vegetation, it underestimates its size relative to the operators’ measurements. In Figure 6B, corresponding to patient 29, a more diffuse infection of the valve can be observed, where the vegetation appears to be composed of regions with differing echogenicities. As in the previous case, although the system correctly localizes the vegetation, it inaccurately defines its boundaries, resulting in an underestimation of its size.

Illustrative examples showcasing the functionality and performance of the algorithm are available at the Supplementary Materials.

## 4. Discussion

AI-based medical imaging analysis has already demonstrated potential to reduce subjective bias, standardize measurements, and improve reproducibility in various domains of cardiovascular imaging [13,14,15,16,17,18,19]. A defining property of AI-based systems is that their behavior is determined entirely by the data on which they are trained. When trained using annotated datasets curated by expert clinicians from multiple institutions, AI systems can effectively emulate a consensus-driven approach. In this manner, the output of the algorithm reflects an aggregated diagnostic perspective, closely approximating the average behavior of diverse specialists. 

Herein, we present a new automatic method to measure vegetation length, showing a concordance with human operators similar to the concordance among them. The AI-based system, however, has the advantage of consistency: it will always measure the same vegetation length when confronted with the same images. This represents a standardized measure, which reduces inter- and intra-operator variability, and will allow a more precise definition of cutoff points and thresholds for clinical practice.

The level of agreement between the measurements obtained by the AI-based system and those recorded by each human operator is comparable to the concordance observed between the operators themselves (Figure 4). Moreover, the concordance coefficient between the AI-based system and the mean of the human measurements reaches 0.74, and falls slightly to 0.71 when the median is used as the reference instead, indicating a high level of agreement that does not depend on the choice of central tendency. The 95% limits of agreement of approximately +6.5 and −8.4 mm are comparable to those observed between human operators. The 95% confidence intervals for inter-operator agreement, as anticipated in the Introduction, are considerably wide, thereby undermining their adequacy as a robust foundation for clinical decision-making. Given that the AI-based system operates as an independent observer, its limits of agreement would be expected to be of similar magnitude. It should be noted that, in addition to inter-operator variability, human operators are also subject to intra-operator variability [8]. In contrast, the AI-based system addresses this limitation by consistently producing the same vegetation length measurement when presented with an identical set of images.

From a practical standpoint, the AI-based system agreed with the surgical decision in 84.7% of cases at the 10 mm threshold and 75.0% at the 15 mm threshold, comparable to the interobserver agreement reported in the literature [8]. In only two cases the system was unable to produce a measurement, because no adequate views were available in both cases. In clinical practice, when no acquisition meets the predefined view criteria, the operator should revert to conventional manual measurement.

We believe that the system described herein holds potential as an assistive tool for echocardiographers, streamlining the measurement of vegetations and enhancing operator confidence, although a multicenter trial is first required to confirm its reliability. Furthermore, it may help simplify the logistical challenges inherent to multicenter studies investigating the role of vegetations in LSIE, potentially obviating the need for centralized core laboratories, given the consistency and reproducibility of its measurements.

Moreover, it is worth mentioning that in a clinical setting there is still a source of variability which cannot be eliminated: image acquisition. Different echocardiographers can still acquire different views of the same vegetation in which different sizes are observed. 

LSIE is a surgical disease with more than 50% of patients needing surgery in the acute phase, which bears a mortality of roughly 30%, higher than most types of cancer. Vegetation size in patients with LSIE is crucial in the decision-making process with some indications of surgery relying only on that parameter and others helping tip the balance in favor of surgery. The decision is not usually based on vegetation diameter alone, but this remains an important factor. The more reliable and reproducible the measurement, therefore, the sounder the decision.

The main limitation of this work is that it is a single-center study (variability across operators in different centers could be higher). A potential multicenter study with many operators would include differences among centers, echo machines, and measurement methodologies. We hypothesize that this context will result in higher interoperator variability, while the AI-based system will still tend towards the average measurement, highlighting the value of consistent AI-based measurements.

Furthermore, measurements of vegetations performed by experts from various research groups have been correlated with embolic events in several studies. A logical next step would be to validate the AI-based system for vegetation size assessment as a potential predictor of embolism. In that scenario, echocardiographers from all centers would have available a universal tool whose measurements are consistent and independent of the operator. It should be emphasized that the present work assesses only the concordance with the vegetation measurements performed by echocardiographers and does not establish superiority or an improved prognostic value. Future studies should focus on improving the prognostic value of the measurement obtained using this algorithm within a real clinical setting. To this end, a multicenter study should be conducted to evaluate the correlation between measurements obtained by physicians using the algorithm and those obtained through the traditional approach, and to assess whether the predictive value afforded by the algorithm is superior to that achieved by physicians alone. Since vegetation size is only one of several determinants of the disease course, such a study should model it jointly with parameters such as vegetation mobility and the causative pathogen, rather than in isolation [4,5,6].

In addition, transformer-based architectures capable of inherently modeling the motion of vegetations within the training process may be of interest, as echocardiographers consider vegetation motion for their identification. This approach was initially discarded due to the challenges associated with annotating vegetations in each individual frame; nevertheless, it may be worthwhile to explore this strategy in future studies. 

Finally, vegetation develops on valves that frequently present pre-existing structural abnormalities such as degenerative thickening or prolapse, calcifications, or annular dilatation, which alter tissue echogenicity and mobility [20,21]. Since the algorithm, like a human operator, must delineate the vegetation against this background, such abnormalities may make the vegetation margins harder to define, or lead non-infective echodense or mobile structures to be mistaken for vegetation tissue. The vegetation itself is a further source of variability, as its morphology differs with the causative pathogen; whether this affects detection and delineation would be worth examining. In this respect, the integration of 3D-TEE imaging represents a promising direction for future research: by providing a more accurate depiction of complex morphologies than 2D-TEE, it may both improve vegetation measurement and help discriminate vegetations from abnormal native or prosthetic tissue.

## 5. Conclusions

In this work, we propose an AI-based system to compute a consistent measure of the vegetation maximal diameter from TEE imaging, reducing the variability inherent to the measurement process: when confronted with the same set of images, the algorithm always outputs the same measurement. The concordance of the AI-based system with human operators is similar to the concordance among human operators. Thus, the AI-based system represents a measurement method in line with current human operators, but more consistent and standardizable. This reduces inter-operator variability and has the potential to allow a more precise definition of cutoff points and thresholds in future clinical guidelines.

## Figures and Tables

**Table 1:** Baseline Characteristics of the patients included.

Age, mean ± standard deviation	66 ± 11
Male	52 (78%)
Diabetes mellitus	16 (24%)
Cancer	9 (13%)
Nosocomial	16 (24%)
*Staphylococcus aureus*	14 (21%)
Coagulase negative staphylococci	4 (6%)
*Streptococcus viridans*	11 (16%)
Enterococci	13 (19%)
Negative cultures	5 (7%)
Mitral	31 (45%)
Aortic	27 (33%)
Multivalvular	9 (13%)
Heart failure	46 (69%)
Renal failure	28 (42%)
Septic shock	16 (24%)
Stroke	14 (21%)
Peripheral embolism	17 (25%)
Surgery	39 (58%)
In-hospital mortality	26 (39%)

**Table 2:** Image-level performance of the three models. Values for the segmentation models correspond to the predicted masks. F1, harmonic mean of precision and recall; IoU, intersection over union; mAP50, mean average precision at an IoU threshold of 0.50; mAP50–95, mean average precision averaged over IoU thresholds from 0.50 to 0.95.

	F1	mAP50	mAP50–95
Vegetation detection	0.73	0.69	0.44
Vegetation segmentation	0.96	0.97	0.75
Valve segmentation	0.53	0.25	0.09

**Table 3:** Classification of the maximum vegetation diameter at the ≥10 mm surgical threshold by the AI-based system and by the mean of the five operators. The analysis is restricted to the 72 vegetations measured by all observers and by the system.

	AI ≥ 10 mm	AI < 10 mm	Total
Operator mean ≥ 10 mm	57	3	60
Operator mean < 10 mm	8	4	12
Total	65	7	72

**Table 4:** Classification of the maximum vegetation diameter at the ≥15 mm threshold by the AI-based system and by the mean of the five operators. The analysis is restricted to the 72 vegetations measured by all observers and by the system.

	AI ≥ 15 mm	AI < 15 mm	Total
Operator mean ≥ 15 mm	26	6	32
Operator mean < 15 mm	12	28	40
Total	38	34	72

**Figure A16:**
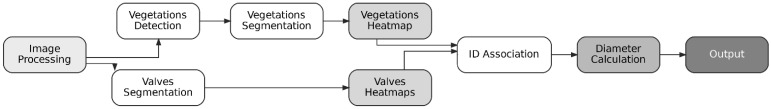
Overview of the system architecture. After a common image-processing stage, the pipeline splits into two branches: the upper one detects and segments vegetations, and the lower one segments the valves; each branch produces its corresponding heatmap. The ID Association block combines both heatmaps to assign a unique identifier to every vegetation across frames, and the Diameter Calculation block derives the vegetation diameter, which is returned as the final output.

**Figure A17:**
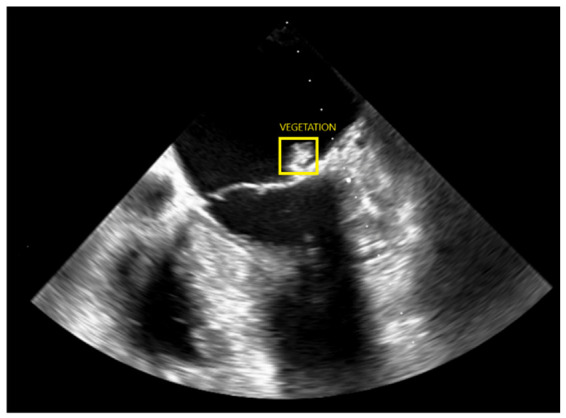
Annotated vegetation in a preprocessed frame of an echocardiogram from a patient diagnosed with endocarditis.

**Figure A18:**
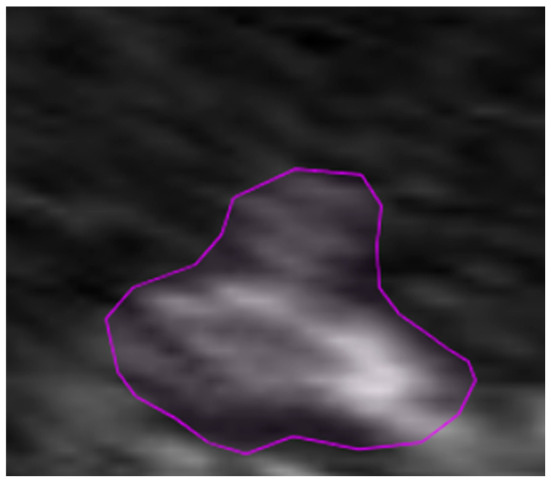
Annotated vegetation in a cropped frame of an echocardiogram from a patient diagnosed with endocarditis.

**Figure A19:**
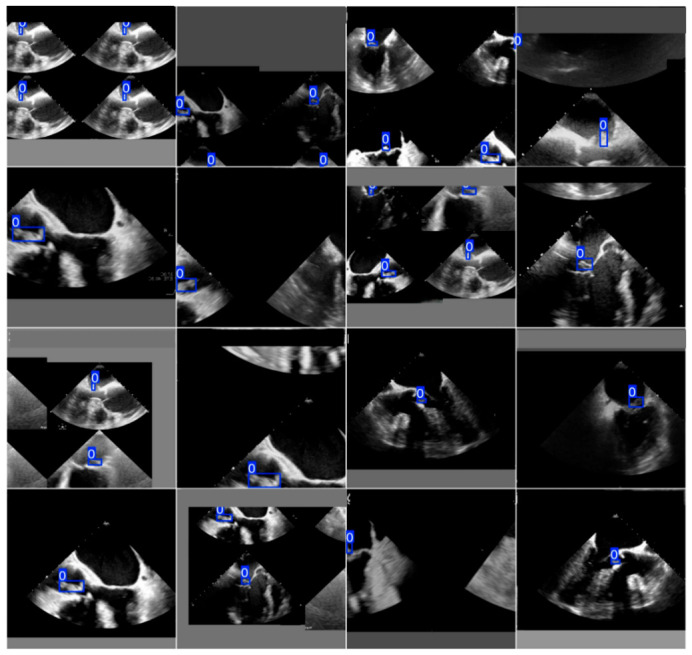
Example of synthetic training samples produced by data augmentation from a single image. This example of an image mosaic was used to train the detection model. Number zero refers to the vegetation label assigned during the training process.

## Data Availability

The data presented in this study are available on request from the corresponding author.

## Supplementary Materials

Illustrative examples showcasing the functionality and performance of the algorithm are available at the following link: https://tinyurl.com/EMBOENDO (accessed on 20 July 2026).

## Abbreviations

The following abbreviations are used in this manuscript:

AI	Artificial Intelligence
LSIE	Left-sided infective endocarditis
TEE	Transesophageal echocardiography
ICC	Intraclass correlation coefficient
CCC	Lin’s concordance correlation coefficient

## Appendix A

**Table A1:** Measurements recorded by the operators for each vegetation. The value “–“ in entries 2 and 41 is due to the fact that the measurements were not performed by the AI, as the available views did not meet the defined criteria.

	Location	Op.1	Op.2	Op.3	Op.4	Op.5	AI
1	Aortic	15	12	19	14	18.6	17
2	Aortic	5	10	6	6	10.4	-
3	Aortic	15	14	13	14.7	14.5	6
4	Aortic	9	11		13	6.6	15
5	Aortic	6	9	9	7.5	13.8	12
6	Aortic	14	19	19	16	15.1	14
7	Aortic	5	10	9	6	8.5	9
8	Aortic	18	24	14	24	23.1	18
9	Aortic	11	12	7	9.7	13.1	11
10	Aortic	22	18	17	18	25.4	21
11	Aortic	15	17	17	19	15.6	14
12	Aortic	10	12	9	13.4	16.2	13
13	Aortic	18	17	18	15.6	18	16
14	Aortic	11	18	16	16.5	12.9	16
15	Aortic	10	12	10	8.4	16	9
16	Aortic	18	19	20	13.6	23.8	22
17	Aortic	12	18	7	12	12.3	17
18	Aortic	10	9	13	12	13	11
19	Aortic	13	10	15	11	10.7	11
20	Aortic	8	8	9	8	12.8	10
21	Aortic	10	16	16	11	16	10
22	Aortic	12	20	11	15.9	13.7	13
23	Aortic	10	7	10	6	9.4	10
24	Aortic	15	14	10	12.5	12.6	12
25	Aortic	18	10	16	20	14.1	11
26	Aortic	9	6	25	24	27.8	15
27	Aortic	21	9	21	16.7	19.6	17
28	Aortic	8	8	8	9.9	11	11
29	Aortic	15	16	17	12	12.4	8
30	Aortic	18	15	19	19	18	13
31	Aortic	30	18	24	18	20	18
32	Aortic	9	11	11	10	10	10
33	Aortic	16	22	13	17	13	21
34	Aortic	11	10	11	16.3	14.4	11
35	Aortic	15	20	14	15	15.2	12
36	Aortic	12	12	6	7.7	9.8	12
37	Mitral	17	17	17	16	20.6	21
38	Mitral	12	13	13	13	14.3	12
39	Mitral	13	11	9	11	12.8	14
40	Mitral	9	3	15	0	8.8	17
41	Mitral	35	34				-
42	Mitral	32	34	30	34	34.1	31
43	Mitral	12	18	12	15	18.9	13
44	Mitral	12	12	15	16	14.5	18
45	Mitral	17	14	9	6	20.9	24
46	Mitral	23	31	23	24	26.3	25
47	Mitral	13	15	20	10	13.6	17
48	Mitral	17	17	21	18	21.7	21
49	Mitral	10	12	12	10	14.9	11
50	Mitral	26	23	26	32.8	27.2	21
51	Mitral	10	8	21	15.8	18.1	10
52	Mitral	12	15	15	13.9	16	10
53	Mitral	14	24	20	17	19.2	19
54	Mitral	10	15	11	12.6	13.7	15
55	Mitral	8	9	9	7	14	16
56	Mitral	20	14	11	13.9	18.9	22
57	Mitral	17	13	14	13	13.8	25
58	Mitral	13	13	15	13.6	16.6	18
59	Mitral	16	18	14	17	19	18
60	Mitral	7	7	8	7.8	6.9	9
61	Mitral	6	5	9	4.3	8.3	6
62	Mitral	16	23	28	16.8	19.4	21
63	Mitral	20	20	11	10	23.7	21
64	Mitral	16	15	15	14.7	15	19
65	Mitral	10	8	6	7.6	15.6	16
66	Mitral	10	11	7	9.5	9.2	9
67	Mitral	24	23	24	25	22.4	24
68	Mitral	17	18	15	20	21.3	26
69	Mitral	13	17	12	13	16.1	17
70	Mitral	25	29	23	23	23.8	28
71	Mitral	9	11	9	9.9	11.1	13
72	Mitral	10	11	12	10	8.8	14
73	Mitral	23	15	12	23	16.5	22
74	Mitral	19	24	21	22.6	21.5	26
75	Mitral	21	31	17	25	28.1	28
76	Mitral	15	12	16	12.7	9.7	15

## Appendix B

Figure A1, Figure A2, Figure A3, Figure A4 and Figure A5 present scatter plots showing the correlation between the measurements obtained by the operators and the AI system, and Bland–Altman plots assessing the agreement between the measurements obtained by both methods.

Figure A6, Figure A7, Figure A8, Figure A9, Figure A10, Figure A11, Figure A12, Figure A13, Figure A14 and Figure A15 present scatter plots showing the correlation between measurements obtained by different operators and Bland–Altman plots assessing the agreement between the measurements obtained by each pair of operators.

## Appendix C

### Appendix C.1. Overview

The proposed algorithm is capable of detecting and measuring multiple vegetations, assigning a unique identifier to each one. To achieve this, various AI models were employed. Prior to being input into these models, all video frames underwent a preprocessing step in which regions outside the echocardiographic cone were removed. This preprocessing was applied consistently both during model training and image annotation. The main objectives were to eliminate any on-screen annotations or patient-identifiable information and to reduce the risk of overfitting by limiting the input to clinically relevant areas only.

The algorithm operates in the following sequence: first, vegetations are detected using an initial AI model that specifies a rectangular region (area of interest, AOI) where a vegetation is present in each frame. These regions are passed to a second AI model responsible for segmentation, where the detailed contour of the vegetation is drawn. Once segmented, the maximum diameter of each vegetation is computed using a rotating caliper method.

Detection and segmentation are also conducted on valve leaflets, using a different set of specifically trained algorithms.

This process is conducted on a frame-by-frame basis. To identify which detections across different frames represent the same structure, a tracking algorithm (BoT-Sort) is applied to the temporal sequence, assigning a unique ID for each vegetation and valve. Then, heatmaps are generated for every structure, and points in these heatmaps are clustered (K-means algorithm, K ranging from 1 to 10, with the optimal number of clusters determined using the elbow method). Each identified cluster is the heatmap of a meaningful structure (vegetation or valve). Finally, to match vegetations with their corresponding valves, Chamfer distances between vegetation and valve heatmaps are calculated.

In the last step, the diameter is calculated for every vegetation detected and every frame. To mitigate the impact of potential spurious detections, the final diameter extracted from each video sequence is computed by averaging the upper half of the measurements derived from each frame. Finally, the vegetation diameter computed for a patient is the maximum across all video sequences processed. Figure A16 illustrates the general workflow of the proposed system.

### Appendix C.2. AI Models

As described in the methodology, three artificial intelligence models were employed, all based on YOLOv12 and YOLOv11 neural networks. Each detection model outputs the coordinates of bounding boxes around potential vegetations, along with an associated confidence score ranging from 0 to 1. This score represents the model’s estimated probability that the identified structure is a true vegetation; higher values indicate greater certainty. For model implementation, a confidence threshold was defined for each network, representing the minimum prediction confidence required to accept a detection. As said in the main manuscript, the thresholds were set at 0.50 for vegetation detection, 0.15 for vegetation segmentation, and 0.31 for valve segmentation.

Increasing this threshold leads to stricter detection criteria, allowing the model to discard uncertain structures that may not represent true vegetations. While this enhances precision, it may reduce recall by excluding some true positives. Therefore, a vegetation candidate is only accepted as a valid detection if its confidence score exceeds the defined threshold.

### Appendix C.3. Data Labeling

Once the dataset is curated, the next step involves the precise labeling of vegetations within each TEE cineloop. This labeling was performed using the Visual Object Tagging Tool (VoTT), an open-source annotation tool that facilitates frame-by-frame labeling of objects within videos. Each frame of the echocardiographic cine loops was examined, and the vegetation was marked with bounding boxes as shown in Figure A17.

To train the vegetation detector, thousands of vegetations need to be labeled and enclosed in bounding boxes. Given the time-consuming nature of the task, an iterative, semi-automatic annotation process was employed. Initially, an AI model was trained using data from the first 149 patients. This model was then applied to a batch of 15 additional patients to generate estimated labels, which were manually corrected by cardiac imaging experts on an image-by-image basis. A new AI model was subsequently trained using the corrected labels. This process was repeated for each successive batch of 15 patients until all 353 patients were annotated. As the model improved with each iteration, the time required for manual labeling progressively decreased.

To construct the training dataset for the vegetation segmentation model, images were extracted from the bounding boxes generated during the vegetation labeling process. The first 15 patients were manually segmented, as illustrated in Figure A18, while the remaining patients were segmented iteratively, following the same approach employed in the generation of the dataset for training the detection model.

To train the valve segmentation model, valve structures were manually annotated in transesophageal echocardiographic frames from 77 patients treated in the cardiology department in whom infective endocarditis had been clinically excluded.

### Appendix C.4. Training Settings

The training set was enhanced using standard data augmentation techniques, such as rotation, scaling, inversion, and mosaic data augmentation, to increase spatial variability in the dataset and improve robustness of the trained model. Figure A19 shows an example of the different versions of an input image generated during this data augmentation process.

For each of the detection and segmentation tasks, a comparative evaluation was conducted among multiple YOLO-based architectural variants, namely YOLOv11n, YOLOv11s, YOLOv12n, and YOLOv12s. Following systematic experimentation, the optimal performance was achieved by the YOLOv12 model for vegetation detection, the YOLOv11-seg model for vegetation segmentation, and the YOLOv11n-seg model for valve segmentation.

Training was conducted over 34 epochs on the training set. An early stopping strategy was implemented to mitigate overfitting, whereby training was halted if no improvement in performance on the independent optimization set was observed for 5 consecutive epochs, and the model weights corresponding to the best-performing epoch were retained. Three distinct loss functions were implemented to evaluate model performance. The val/box_loss metric quantifies the localization loss related to bounding box predictions. On the other hand, the val/cls_loss metric quantifies the classification loss associated with detected objects in the images. Finally, the val/dfl_loss metric represents the Distribution Focal Loss, which assesses the model’s capability to predict fine-grained anchor distributions.

Unlike traditional methods that augment datasets by generating additional samples in advance, a different (random) augmented dataset is generated at each training epoch. This approach enhances data diversity and mitigates the risk of overfitting. Among all trained models, the YOLOv12 detection model (9.3 million parameters) proved to be the most challenging to train. The training hyperparameters employed for this model were as follows: The model weights were initialized using the pretrained weights provided by Ultralytics, and no layers were frozen during the training process. AdamW optimizer was employed with a learning rate of 0.002 and a final learning rate of 1% of the initial learning rate, with 3 warmup epochs, a momentum of 0.9, and a weight decay of 0.001. The mosaic data augmentation was disabled at epoch number 20, the batch size was set to 64, and the input image size was 640 × 640 pixels in the detection models and 320 × 320 in the segmentation models.

## Appendix D

Table A2 reports the agreement between every pair of observers, including the AI-based system, in the classification of vegetations into the three surgical decision categories. Overall agreement ranged from 0.583 to 0.681 among the operators and from 0.542 to 0.667 between the operators and the system, and the corresponding quadratic-weighted κ ranged from 0.475 to 0.740 and from 0.378 to 0.541.

**Table A2:** Pairwise agreement between observers in the classification of the maximum vegetation diameter into the three surgical decision categories (<10 mm, 10–14.9 mm and ≥15 mm). Overall agreement is the proportion of vegetations assigned to the same category by both observers.

Comparison	Overall Agreement	Weighted κ	Positive Agreement		
			<10 mm	10–14.9 mm	≥15 mm
Op. 1 vs. Op. 2	0.625	0.605	0.636	0.500	0.714
Op. 1 vs. Op. 3	0.583	0.561	0.615	0.440	0.676
Op. 1 vs. Op. 4	0.625	0.586	0.640	0.528	0.697
Op. 1 vs Op. 5	0.597	0.502	0.333	0.491	0.740
Op. 1 vs AI	0.653	0.459	0.353	0.618	0.750
Op. 2 vs Op. 3	0.583	0.512	0.500	0.435	0.714
Op. 2 vs Op. 4	0.667	0.606	0.593	0.571	0.765
Op. 2 vs Op. 5	0.625	0.475	0.300	0.531	0.773
Op. 2 vs AI	0.583	0.378	0.211	0.510	0.730
Op. 3 vs Op. 4	0.681	0.740	0.839	0.511	0.727
Op. 3 vs Op. 5	0.597	0.497	0.417	0.468	0.740
Op. 3 vs AI	0.556	0.409	0.348	0.449	0.694
Op. 4 vs Op. 5	0.625	0.546	0.522	0.520	0.732
Op. 4 vs AI	0.542	0.457	0.455	0.423	0.657
Op. 5 vs AI	0.667	0.541	0.533	0.577	0.753

The quadratic-weighted κ accounts for the ordinal nature of the classification and penalizes larger disagreements more heavily. Because κ is sensitive to category prevalence, the positive (specific) agreement is reported separately for each category. The analysis was restricted to the vegetations measured by all observers and by the system, so that every comparison is based on the same cases.
